# Short Communication: Lived experience perspectives on genetic testing for a rare eye disease

**DOI:** 10.1007/s12687-023-00677-5

**Published:** 2023-10-03

**Authors:** Mallorie T. Tam, Alonso Daboub, Hayami Lou, Julie M. Robillard

**Affiliations:** 1 Department of Medicine, Division of Neurology, University of British Columbia, Vancouver, BC Canada; 2 BC Children’s and Women’s Hospital, Vancouver, BC Canada

**Keywords:** Aniridia, Rare eye disease, Genetic test, Decision-making, Lived experience

## Abstract

This qualitative study explored the motivators and barriers for genetic testing for individuals with aniridia. Semi-structured interviews were conducted with 8 participants. The main findings highlighted the complex and interrelated factors involved in the decision-making process, including family planning, learning about the specific pathogenic variant of the disease and having access to genetic testing. Benefits and potential risks of genetic testing for aniridia were also discussed. For participants, gaining knowledge about their condition was perceived as a benefit, while administrative issues and concerns around privacy were identified as risks. Increased access to quality information about genetic testing and to the service and associated resources are needed to better support people living with aniridia.

**Supplementary Information:**

The online version contains supplementary material available at 10.1007/s12687-023-00677-5.

## Introduction

Aniridia is a congenital eye condition characterized by a complete or partial absence of the iris, resulting in pupil abnormalities and reduced visual acuity. This rare disease occurs in 1 in 50,000 to 100,000 births worldwide, with some symptoms commonly present at birth and others arising over time (“Aniridia: MedlinePlus Genetics” 2009; Blanco-Kelly et al. 2013). Individuals with aniridia experience eye pain, sensitivity to light, and can develop other ocular issues such as cataracts and glaucoma later in life.

Most aniridia cases have been found to originate from a pathogenic variant in the *PAX6* gene, characterized by an absence of the iris. It occurs as either an isolated ocular abnormality or as part of the Wilms-tumour-aniridia-genital anomalies-retardation (WAGR) syndrome, caused by a deletion in regions including *PAX6* and *WT1*. Individuals with WAGR syndrome are at a higher risk of developing Wilm’s tumour, a rare kidney cancer mainly affecting children (Moosajee et al. 2018). Recent literature has shown that other genes such as *PITX2*, *FOXC1*, *CYP1B1* and *FOXE3* are also implicated in aniridia and iris anomalies, contributing to the level of genetic complexity of the disease (Daruich et al. 2023). While aniridia begins with a clinical diagnosis, a genetic investigation can assess family history, confirm the diagnosis and inform eligibility in different clinical trials (Richardson et al. 2016).

Given that approximately two-thirds of aniridia cases are familial with autosomal dominant inheritance and one-third are sporadic (Richardson et al. 2016; Lee et al. 2008), *PAX6* gene sequencing (referred to as genetic testing for the remainder of this paper) plays a significant role in the diagnosis and management of the disease. Genetic tests can benefit individuals with aniridia by providing diagnostic validation, family risk assessment and informed treatment and counselling plans. Genetic testing is especially critical in cases that are deemed sporadic, where the risk of developing Wilm’s tumour needs to be considered (Wiggs and Pierce 2013). The decision to undergo genetic testing is notably significant, particularly among adult individuals living with aniridia. For adults bearing this condition, genetic testing serves as an important tool for determining the likelihood of transmitting the pathogenic variant to their offspring, thus guiding their choices around family planning. Furthermore, for those adults who have already become parents to children with aniridia, genetic testing offers insights into the syndromic nature of their case and the risk of Wilm’s tumour development. However, given the crucial decision-making that adults with aniridia face, there is limited exploration of the perspectives of these groups. To address this gap, this study aims to understand the motivators and barriers for genetic testing for individuals with aniridia.

## Methods

Participants were recruited from aniridia communities on Facebook, an in-person conference, and through snowball sampling between October 2021 and September 2022. Eligible participants had to be over the age of 18 years, able to read, understand and speak English, and self-report a diagnosis of aniridia. Interview guides were developed and piloted by the research team and questions focused on the lived experiences and perspectives of people with aniridia. Questions were focused on participants’ experiences with (1) genetic testing for aniridia; (2) genetic counselling; (3) receiving genetic test results and (4) aniridia research. Examples of interview questions can be found in Supplementary Table 1. The interview transcripts were analyzed qualitatively using the NVivo software. We conducted a descriptive content analysis of the transcripts to uncover emerging themes around genetic testing for aniridia. A preliminary coding guide was developed and applied to an initial set of interviews (*n* = 2) by two independent coders (MTT and AD). The coding guide was refined through an iterative process by discussing and resolving discrepancies until an agreement score over 80% was reached. The final coding guide was applied to the remaining sample of interview transcripts.

## Results

We conducted 8 semi-structured interviews (6 females, 2 males) with individuals with aniridia from Canada and the Philippines. Participant ages ranged from 19 to 55 at the time of the interview. All participants had a clinical diagnosis of aniridia, and five participants did not receive genetic testing. Three participants also had a family history of aniridia. Details of the participants are summarized in Table 1.

**Table 1:** Summary of participant details and perspectives on genetic testing

Participant	Age at the time of interview	Sex	Genetic testing status	Family history of aniridia	Reasons for genetic testing	Perceived barriers to genetic testing	Perceived benefits of genetic testing
1	44	Female	Completed	Yes	Family planning	Readiness to undergo genetic testing process	Having a game plan after receiving results
2	55	Female	Not completed	No	Learn about other health conditions associated with pathogenic variant	Concerns around stigma and logistical errors during testing (e.g. mixed-up samples)	Learning more about the disease
3	24	Female	Completed	No	Learn about specific pathogenic variant, associated complications, and treatment options	The cost of genetic testing	Learn about specific pathogenic variant, associated complications and treatment options
4	38	Female	Not completed	No	Learn about specific pathogenic variant, treatment options, and to help plan the future of family	The cost of genetic testing	Learn about specific pathogenic variant and treatment options
5	26	Female	Not completed	No	Does not feel the need to get genetic testing done	Not receiving enough information about aniridia to pursue genetic testing	Better understanding of one’s condition and impacts on personal health
6	39	Female	Completed	No	Family planning; learning whether offspring will have aniridia	No mention of barriers	Reassurance of knowing whether children will have aniridia or not
7	45	Male	Not completed	Yes	Learning about treatment options	Lack of information about genetic testing	Helping future generations in the family and contribute to research to advance knowledge about prevention and treatments
8	19	Male	Not completed	Yes	No interest in genetic testing	Concerns around logistical errors during and after testing; lack of information about genetic testing	Potential to treat personal experiences with aniridia

For participants who decided to undergo genetic testing (*n* = 3), several factors were involved in the decision-making process. Planning to start a family was an important factor for all participants, and all shared that genetic testing was a factor for reproductive decision-making. Learning about their specific pathogenic variant also meant knowing the potential risks for other personal health issues (2/3 participants). One participant explained, “In my view, [genetic testing] is better understanding your condition and what you may have in the future with other conditions.” Genetic testing was also described as a factor to understand the different treatment options that were available to participants. One participant emphasized that being emotionally ready to undergo the entire genetic testing process, including receiving the results, was a key factor that they had to take into account: “If you’re wishy-washy about [genetic testing], it can be really stressful if you’re faced with making a decision before you do that procedure. You should already have your decision made, do the procedure, get your answer and then follow through on your next steps.”

For participants who did not receive genetic testing (*n* = 5), access in terms of obtaining enough information, knowing where to receive testing and the availability of testing were important factors that played a role in their decision (3/5 participants). Financial considerations regarding the cost of testing were also raised as a factor in decision-making (4/5 participants). Two individuals expressed their interest in undergoing genetic testing, explaining their wish to help their family’s future generations with aniridia and advancing knowledge around treatment and prevention. One participant expressed his willingness to undergo genetic testing, but cited insufficient information as a barrier to proceeding with testing.

From the participants’ perspectives, the benefits of genetic testing were focused around knowledge. More specifically, there was expressed value in gaining knowledge about their specific pathogenic variant and other associated health conditions that they needed to be aware of (4/8 participants). For two participants, genetic testing meant knowing what they were dealing with so that it could help them find a solution to their problems and plan for their future. From another participant’s viewpoint, genetic testing advances the knowledge about the *PAX6* gene in hopes of helping future generations, preventing sporadic aniridia and discovering treatments.

Perceived risks around genetic testing for aniridia involved administrative and logistical issues, such as privacy around storing an individual’s test results or mislabelling test tubes (2/8 participants). One participant raised concerns around stigma, being given a label as being “aniridic” which caused negative emotions in managing the disease: “I find it really frustrating because I didn’t grow up with [aniridia] or know that I have it. The term ‘aniridic’, I’m not an aniridic. I’m somebody that has aniridia but I’m not an aniridic. I don’t like that at all.” Other risks pertained to passing the disease on to the participants’ children and testing during pregnancy (2/8 participants).

## Discussion

By engaging people with lived experiences of aniridia, this study revealed several motivators and barriers to genetic testing. Reproductive planning was cited as an important factor influencing the decision-making process of the study’s participants. This finding resonates with a recent study exploring the experiences of individuals with intellectual disabilities, where they identified potential benefits of genetic testing as gaining a better understanding of their condition, ruling out other conditions and improved access to relevant support (Strnadová et al. 2023). A participant in the current study conveyed a desire to undergo genetic testing and participate in research to contribute to advancing knowledge that could alleviate the burden of the condition for future generations. This motivation is consistent for other rare disease patients, whereby altruism is a strong catalyst for pursuing genetic testing, particularly in the context of research (Dwyer et al. 2022).

The cost associated with genetic testing emerged as a notable barrier for some study participants. This concern aligns with the findings from a qualitative study conducted in Boston, USA, where individuals affected by rare diseases identified the cost as the primary barrier preventing them from receiving genetic testing, with specific concerns around insurance coverage (Dwyer et al. 2022). Similarly in an Australian discrete choice experiment study, participants reported a reluctance to genetic testing when presented with higher costs and requirement to disclose results to health insurers (Goranitis et al. 2020). It is important to account for the geographical location of patients, as the costs and insurance coverage can vary (Robillard et al. 2021).

The importance of receiving accurate information about genetic testing emerged as crucial factors guiding participants’ decision-making. This aspect is reflected by a participant in this study who was deterred from pursuing genetic testing due to a lack of information. This finding is especially relevant in light of previous studies that have found a lack of information and guidance on accessibility and reimbursement for genetic testing (Robillard et al. 2021; Chiang et al. 2015). Individuals with other inherited retinal diseases share similar needs for comprehensive information about gene therapy (Mack et al. 2023). Despite knowledge gaps, these individuals reported willingness to undergo treatment if it were available (Mack et al. 2023). Given the complexity of genetic testing and its implications, as well as the continuous development of new technologies, it is critical that individuals with rare genetic conditions are informed on the potential benefits and risks, and updated information around genetic testing and therapy (Robillard et al. 2021; Mack et al. 2023). Moreover, as genetic testing can impact eligibility for participation in research and clinical trials, information about these implications should be accurately disseminated to individuals with aniridia to avoid confusion or coercion, as well as to mitigate therapeutic misconception (Benjaminy et al. 2015).

When thinking about the effective delivery of genetic testing information and knowledge, genetic counsellors play a crucial role in supporting individuals navigate their ocular disease (Gillespie et al. 2014). While access to genetic counselling should be a priority to support decision-making around testing and specifically access to genetic counsellors with expertise in rare diseases, the use of online decisional support could be a potential measure to mitigate the shortage of genetic counsellors for rare diseases (Dwyer et al. 2022). These tools can also serve to raise awareness of genetic testing and counselling options and increase access to information and support for patients and families (Dwyer et al. 2022).

The small sample in this study is an important limitation, and while data saturation was achieved with our data set, we acknowledge that a larger and more diverse sample may have yielded different perspectives. Future studies can expand upon the present work by engaging a broader demographic of individuals living with aniridia, and by including a focus on parents of children with aniridia undergoing the decision-making process for genetic testing. In addition, we acknowledge that aniridia can manifest due to numerous pathogenic variants. Given that our study did not include inquiries into the specific pathogenic variants present in participants, it is important to interpret the findings accordingly.

## Conclusion

This study offers insights into the motivations and barriers associated with genetic testing from the perspectives of individuals living with aniridia. The present findings emphasize the need to engage with people living with aniridia throughout their patient journey to gain an understanding of their needs and the factors influencing their decisions regarding genetic testing. Future studies should build upon this research, specifically investigating the factors that influence decision-making among parents of children with aniridia, in effort to improve the quality of life for individuals impacted by this condition.

### Supplementary information

ESM 1 (DOCX 15 kb)
