# Supplementary material for: Short Communication: Lived experience perspectives on genetic testing for a rare eye disease
Source: J Community Genet. 2023 Oct 3;15(1):97–101. doi: 10.1007/s12687-023-00677-5 (PMC10857987; doi:10.1007/s12687-023-00677-5)
Supplement: Supplementary file 1 — (DOCX 15 kb) [file 12687_2023_677_MOESM1_ESM.docx]

Supplementary Table 1. Examples of interview themes and questions.

| **Genetic Testing for Aniridia** |
| --- |
| - Tell me about your decision to undergo genetic testing. - When did you receive genetic testing? - Can you describe your genetic testing experience? - Have you considered undergoing genetic testing for aniridia? - Has anyone provided you information about genetic testing? - Tell me about your decision not to undergo genetic testing? - In your view, what are some of the benefits of undergoing genetic testing? - In your view, what are some of the risks of undergoing genetic testing? |
| **Genetic Counselling Experience** |
| - Tell me about your experience with genetic counselling. - If you did not receive genetic counselling, what made you decide not to get genetic counselling? - What other forms of support were important throughout the genetic testing process? - What services or resources did you seek out before undergoing genetic testing? - Is there anything you wish you had more support with? - Have you received or were you offered genetic counselling with considering genetic testing? |
| **Receiving Genetic Test Results** |
| - Who shared and reviewed your test results with you? - Were there any aspects of the test results that were unclear? - What other details do you with the genetic test was able to tell you about your condition? - Did receiving your genetic test results have any impact on your family? |
| **Aniridia Research** |
| - Have you previously participated in research that recruited for individuals with aniridia? - Has genetic testing been offered to you as part of these types of research studies? |
